# Clinical evaluation for chin augmentation: literature review and algorithm proposal^☆^

**DOI:** 10.1016/j.bjorl.2015.09.009

**Published:** 2016-01-07

**Authors:** Helena Hotz Arroyo, Isabela Peixoto Olivetti, Leila Freire Rego Lima, José Roberto Parisi Jurado

**Affiliations:** a Universidade de São Paulo (USP), Departamento de Otorrinolaringologia, São Paulo, SP, Brazil; b Instituto Jurado de Educação e Pesquisa, São Paulo, SP, Brazil

**Keywords:** Genioplasty, Chin, Plastic surgery, Maxillofacial development, Jaw, Mentoplastia, Queixo, Cirurgia plástica, Desenvolvimento maxilo-facial, Mandíbula

## Abstract

**Introduction:**

The esthetic balance of the face results from harmonic and symmetrical facial proportions. The literature describes several methods for lower-third facial analysis, but lacks a simple and practical method.

**Objective:**

To review the methods of analysis of the ideal projections of the chin based on soft tissues, showing the advantages and disadvantages of each.

**Methods:**

Literature review through the PubMed database.

**Results:**

The following methods for chin analysis based on soft tissues were reviewed: Gonzalles-Ulloa, Goode, Merrifield, Silver, Legan, Gibson & Calhoun, cervicomentual angle, and mentocervical angle.

**Conclusion:**

An adequate analysis of the proportions of the face and facial disharmony is essential for the correct indication of the necessary procedures and good surgical outcome. The authors propose an algorithm to facilitate the indication for chin augmentation surgery.

## Resumo

**Introdução:**

O equilíbrio estético da face resulta de proporções faciais harmônicas e simétricas. A literatura descreve vários métodos de análise do terço inferior da face, mas carece de um método simples e prático.

**Objetivo:**

Revisar os métodos de análise da projeção ideal do mento baseadas em tecidos moles, mostrando as vantagens e desvantagens de cada um.

**Método:**

Revisão da literatura através da base de dados Pubmed.

**Resultados:**

Os seguintes métodos para análise do mento baseada em partes moles são revisados: Gonzalles-Ulloa, Goode, Merrifield, Silver, Legan, Gibson & Calhoun, ângulo cervicomentual e ângulo mentocervical.

**Conclusão:**

A análise adequada das proporções da face e desarmonia facial faz-se essencial para a correta indicação dos procedimentos necessários e bom resultado cirúrgico. Propomos um algoritmo para facilitar indicação de avanço de mento.

## Introduction

Facial harmony has been studied for centuries; by the ancient Greek philosophers who tried to uncover the beauty of the elements, by the Egyptian sculptors with their complex facial harmony, and by the Renaissance artists, such as Michelangelo and Leonardo Da Vinci, who sought concrete measures for facial proportions.^1^, ^2^ These legacies have contributed to our current knowledge of applied facial esthetics. Surgeons must know the ideal proportions of the face to correctly indicate procedures to their patients, as an incorrect analysis leads to inappropriate conclusions.^2^, ^3^

The mid-third, especially the nose, receives greater attention as it is the most prominent part of the face. On the other hand, the lower third should be taken into account, since a small or retracted chin results in facial disharmony, especially when analyzing the profile.^3^ Such disproportion can cause the patient to misinterpret the nose projection, believing it to be larger than it actually is, and to seek a rhinoplasty procedure to repair the facial disharmony.^4^, ^5^ It is the responsibility of the surgeons to esthetically evaluate the face as a whole, analyzing the facial proportions and to decide what procedure or procedures can benefit their patients.^3^, ^6^, ^7^

In this context, the lower third (lips and chin) should not be overlooked, as it can have a significant impact on the profile, postoperatively.^7^ The initial evaluation of the lower third of the face must identify a retropositioned chin and rule out mandibular dimorphism – such as micrognathia (vertical and horizontal mandibular hypoplasia) and retrognathia (retracted mandible relative to the maxilla) – that are associated with dental occlusion abnormalities, most commonly Angle class II dental malocclusion. These cases require cephalometric analysis for possible programming of orthognathic surgery.^8^

Patients with such deformities who refuse more extensive procedures may be submitted to chin augmentation; however, they should be aware of its limitations in improving facial profile and occlusion.^3^, ^7^, ^9^ Nonetheless, it is not unusual for candidates for chin augmentation to have underdevelopment of the mandibular symphysis (horizontal microgenia – the presence of normal vertical height, with retracted bone portion), but with normal occlusion (Angle class I). These patients may benefit from this procedure alone.^8^, ^9^

There are several described methods to analyze the ideal chin projection based on soft tissue, each with its particularities, but none of them complete or ideal.^7^ This article aims to systematically review such methods, showing the advantages and disadvantages of each method in a simple and practical manner. Subsequently, the authors propose a clinical evaluation algorithm for chin augmentation indication.

## Methods

A literature review was conducted using the PubMed database, from 1992 to April 2015. The authors selected articles in English and Spanish related to clinical evaluation for chin augmentation using the following words: analysis and augmentation mentoplasty (four articles), clinical analysis and genioplasty (22 articles), clinical analysis and chin augmentation (21 articles), chin position and profile analysis (46 articles).

This review included only articles that mentioned the methods used to analyze the lower third of the adult face based on photographic documentation of patients (19 articles). It excluded those that exclusively discussed cephalometric analysis through radiography; discussions on Angle class III; analyses of patients with sleep apnea or malformations; articles related to dental extractions and orthodontic devices; evaluations through computed tomography; ethnic studies or studies in children; and descriptions of surgical techniques.

Subsequently, the lists of references of the selected articles were analyzed and the most significant were included in the review, especially those of a historical nature, even dated prior to 1992.

## Results

The most relevant methods for the analysis of facial proportion based on soft tissues, and thus, through photographs, are reviewed below.

**Figure 1:**
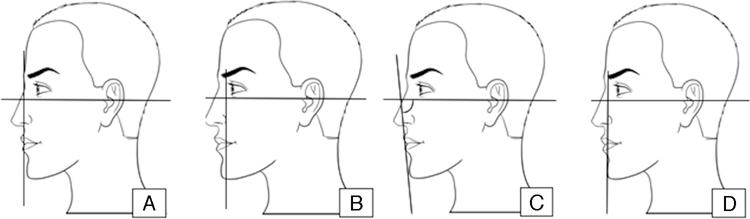
A, Gonzalles-Ulloa; B, Goode; C, Merrifield; D, Silver.

Gonzallez-Ulloa traced a line perpendicular to the horizontal line of Frankfort and tangential to the nasion (point of deepest nasal root depression, in the midline), called the zero meridian (Fig. 1A). He proposed that, in a face with ideal proportions, the pogonion (the most prominent point of the chin) should be on that line or immediately posterior to it. He classified chin retropositioning as grade I, less than 1 cm posterior to the meridian, grade II between 1 and 2 cm, and grade III more than 2 cm.^1^

Goode traced a line perpendicular to the horizontal line of Frankfort passing through the alar groove (Fig. 1B). This method proposes that the pogonion must be on that line or immediately posterior to it.^10^

Merrifield's Z angle is formed at the lower intersection between the horizontal line of Frankfort and another traced between the pogonion and the most protruding region of the lips (Fig. 1C). Its ideal value must be between 75° and 85° (80° ± 5°).^11^

In the method proposed by Silver, a line is traced perpendicular to the horizontal line of Frankfort tangential to the border of the mucocutaneous transition of the lower lip (Fig. 1D). The pogonion must be in this line or up to 2 mm behind – as preferred in women (Fig. 1).^7^

**Figure 2:**
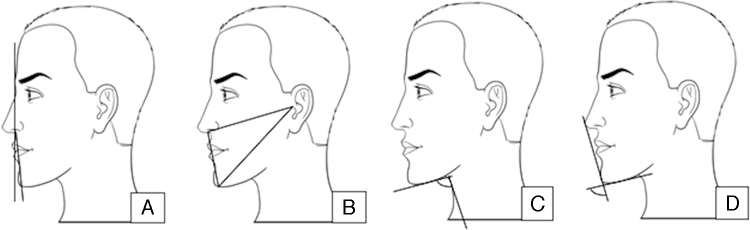
A, Legan; B, Gibson & Calhoun; C, Cervicomental angle; D, Mentocervical angle.

Legan proposed an “ideal” angle to evaluate facial convexity. Legan's angle is measured between a line traced from the glabella to the subnasal point and another from the subnasal point to the pogonion (Fig. 2A). An optimal value of 12° is suggested, which may vary from 8° to 16°.^7^, ^10^, ^12^

The inferior face triangle was proposed by Gibson & Calhoun (Fig. 2B). It is defined by three points: the tragus (T), the subnasal point (S), and the point of chin definition (C). Point C is the intersection of an arc centered in T that is tangential to the chin. The SC line and the T angle reflect the height of the lower third of the face. It proposes as ideal measures a TC/TS ratio of 1.15 to 1.19 and the angle S from 88° to 93°.^10^

The cervicomental angle (CMA) is formed by a line that is tangential to the submental point – from the chin to the subcervical region – and another tangential to the neck at the subcervical region intersection – the lowest point between the submental area and the neck (Fig. 2C). The ideal CMA is described as 121° for men and 126° for women.^3^

The mentocervical angle (MCA) has two definitions. According to Lehmann, the MCA is determined by a line from the nasal tip to the pogonion that crosses the line that is tangential to the submental point, with normal values varying from 110° to 120°. Powell and Humphreys defined the MCA as a line intersection from the glabella to the pogonion with another tangential to the submental area (starting from the subcervical region). Thus, the MCA includes analysis of the nasal tip, neck position, and chin projection (Fig. 2D). This angle increases with the increase in nasal projection and *vice versa*.^3^

## Discussion

The photographic analysis of a patient's profile has limitations regarding the understanding of the craniofacial morphology, as the correlation between soft tissues and bony parts are not proportional, and soft tissue growth is practically independent of skeletal development.^11^ Additionally, some variables are difficult to measure statically, in two dimensions. For instance, the evaluation of the latero-lateral diameter of the chin and analysis of facial harmony in the frontal view are hindered, as well as the dynamic smile view. In the future, three-dimensional analysis and videographies will become more accessible and will be very useful. At the moment, the cost is still a major barrier to these methods, and photographic analysis remains very useful, simple, and practical, as it is inexpensive, does not expose the patient to radiation, and allows good assessment of structures.^3^, ^13^, ^14^

Of the analyzed methods, four of them (zero meridian of Gonzalles-Ulloa, Goode's technique, Merrifield's Z angle, and Silver's technique) use the horizontal line of Frankfort, traced from the upper border of the external auditory canal to the inferior orbital rim. However, the photographic analysis cannot precisely determine the inferior orbital rim, as it is a bone reference point. Gonzalez-Ulloa suggests that, to attain the identification of the Frankfort line, the change of light that usually appears between the lower eyelid and the cheek should be used as a parameter (Table 1).^1^ Thus, the use of these methods in photographic analysis can lead to inaccuracies and interobserver variations. The authors therefore suggest applying these methods when the inferior orbital rim is easily identified in the photograph or by direct patient analysis.

**Table 1:** Comparison of the main methods for chin position analysis.

Method of analysis	Description	Ideal values	Advantages	Disadvantages
Gonzalles-Ulloa – zero meridian	Line perpendicular to Frankfort line passing through the nasion	Pogonion in line or right posterior to it	Simple	Depends on the Frankfort line; varies with nasion
Goode – perpendicular alar	Line perpendicular to Frankfort line going through the alar groove	Pogonion in line or right posterior to it	Simple	Modified with the size of the alar base; depends on the Frankfort line
Merrifield angle Z	Angle between the line of Frankfort and a line drawn between the pogonion and most protuberant lip region	Between 75° and 85°	Analysis based on soft tissues	Depends on the Frankfort line
Legan – angle of facial convexity	Angle formed between the line from the glabella to the subnasal point and another from the subnasal point to the pogonion	Between 8° and 16° – ideally 12°	Analysis based on soft tissues	Modified with maxillary hypoplasia
Facial triangle of Gibson & Calhoun	Triangle formed between the tragus (T), the subnasal point (S) and the point of chin definition (C)	TC/TS from 1.15 to 1.19 and the S angle from 88° to 93°	Analysis based on soft tissues	Requires calculations
Silver	Line perpendicular to the Frankfort line, tangential to the mucocutaneous transition of the lower lip	Pogonion in line or 2 mm behind	Simple	Very comprehensive; depends on the Frankfort line
Cervicomental angle	One submental line and one that is tangential to the neck at the subcervical region intersection	121° – ♂ 126° – ♀	Analysis based on soft tissues	It is modified with the subcutaneous neck tissue
Mentocervical angle	From the nasal tip to the pogonion, crossing the submental line	110°–120°	Integrates the nasal tip analysis, neck position and chin projection	It is modified with the nasal tip

Ahmed et al. evaluated the difference between four chin assessment methods – Silver, Gonzalles-Ulloa, Legan, and Merrifield – by analyzing 100 photos of patients undergoing rhinoplasty. They found that, depending on the method of analysis, the number of patients with microgenia ranged from 17% to 62% of men and 42% to 81% of women. The most conservative method was Legan's method (17% of men and 42% of women), whereas the one that included the most patients with microgenia was Silver's method (62% of men and 81% of women). In total, 21% and 58% of men and women, respectively, were included in three or more criteria.^7^

One should bear in mind that, if the analysis of the chin position points to its retropositioning, a cephalometric assessment to rule out mandibular dimorphism must be performed.

In general, chin augmentation is attained through genioplasty techniques (with increase in the vertical or horizontal plane or both); fillers (homologous and autologous), or alloplastic implants (with higher gain in the horizontal plane).^6^ Therefore, the assessment of the vertical height of the chin and position of the lower lip complement the profile analysis and assist in the choice of technique.^4^, ^15^, ^16^

**Figure 3:**
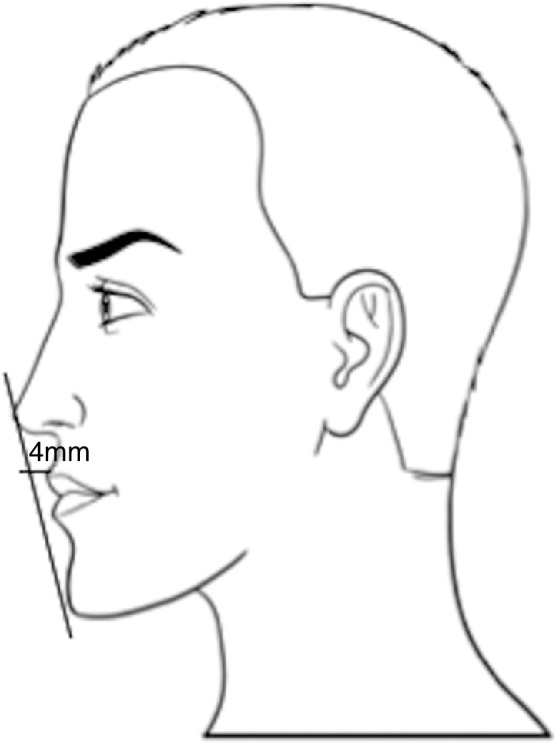
Rickett's E-line.

One method to assess the chin vertical height determines the ratio between the distances from the subnasal point-upper lip and chin-lower lip, which should be 1:2.^6^, ^7^ The position of the lips in relation to the nose and chin was described by Ricketts through the E-line, which is traced from the highest point of the nasal tip (pronasali) to the most prominent portion of the chin (pogonion).^6^, ^17^ The E-pass line should be at 4 mm from the upper lip and at 2 mm from the lower lip (Fig. 3).^18^

Finally, the indication for chin augmentation – after ruling out mandibular dimorphism – depends on the methods used to calculate facial proportions as a whole. The procedure can be performed alone or combined with rhinoplasty, rhytidoplasty, or submental liposuction.^19^

Considering all the different methods of analysis, the authors suggest using the three with which the surgeon is most familiar, and applying them routinely. The procedure is indicated when the patient meets at least two microgenia criteria. Such measures are only guidelines, as one must consider the surgeon's experience and above all, the patient's expectations, respecting their ethnic characteristics and overall conditions (age, gender, comorbidities) (Table 2).^8^

**Table 2:** Chin augmentation indication.

Analysis of the chin position (by at least three different methods, two positive for microgenia)
Analysis of the chin vertical height
Surgeon's experience/available techniques
Patient's expectation/regional aspects

**Figure 4:**
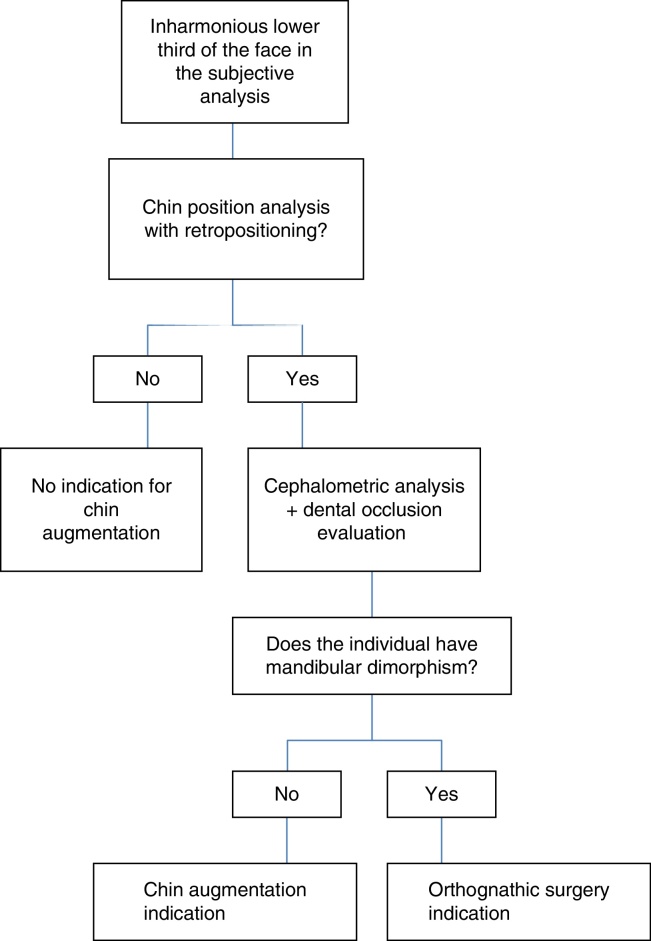
Algorithm for chin augmentation indication.

Therefore, the authors put forth an algorithm to evaluate the lower third of the face, as a proposal for chin augmentation indication (Fig. 4).

## Conclusion

Of the various methods used to analyze chin projection, none seems ideal by itself. Thus, to identify the patient's mandibular deformity, the authors suggest the association of methods, considering the surgeon's experience, available techniques, and patient's expectations (Table 2). An adequate analysis of facial proportions and disharmony is essential for the surgeon to correctly select the appropriate procedure for the patient and, thus, to attain a good surgical outcome.

## Conflicts of interest

The authors declare no conflicts of interest.
